# A57 ULCERATIVE COLITIS DISEASE ACTIVITY IS DOMINATED BY INNATE IMMUNITY AND FEATURES OF TISSUE REMODELING

**DOI:** 10.1093/jcag/gwab049.056

**Published:** 2022-02-21

**Authors:** K Madill-Thomsen, J Venner, S Pon, K Kroeker, F Peerani, L A Dieleman, K Wong, D C Baugmart, P Halloran, B Halloran

**Affiliations:** 1 University of Alberta Faculty of Medicine & Dentistry, Edmonton, AB, Canada; 2 University of Manitoba, Winnipeg, MB, Canada

## Abstract

**Background:**

Ulcerative colitis (UC) is a chronic inflammatory condition affecting the colonic epithelium, with potential roles for the inflammasome, complement activation, T cells, and the microbiome in pathogenesis. We applied an established method of microarray-based gene expression analysis to a set of 128 UC biopsies (from 113 patients), to elucidate the molecular changes associated with active UC.

**Aims:**

Our aim was to describe the molecules most associated with UC disease activity (the endoscopic Mayo score) and to annotate these molecules into biological processes.

**Methods:**

128 UC colonic biopsies were collected at the University of Alberta Hospital (Edmonton, Alberta) and Cedars-Sinai Hospital (Los Angeles, California) during standard of care colonoscopy. Biopsies were processed using Affymetrix microarrays. Gene expression data from the population was visualized using volcano plots (showing fold change and association between genes and endoscopic Mayo score), and heatmaps (showing expression of the top 30 genes in a previously established cell panel). Overexpression of top genes was analyzed using Gene Ontology and KEGG pathways.

**Results:**

The volcano plot (**Figure 1A**) showed strong associations between the endoscopic Mayo score and components of innate immunity, e.g. complement factor B (CFB), C1-inhibitor (also known as SERPING1), chitinase 3-like 1 (CHI3L1), and inflammasome genes (ZBP1 and PIM2). Moderate associations with calprotectin (S100A8 and S100A9), other inflammasome components (CASP1 and NLRP3), and T cell transcripts (i.e. CTLA4, PDL1) were observed. Targets of biologic therapy (TNFA, ITGA4/B7, IL12B) were weakly associated with the endoscopic Mayo score.

Expression of the top genes in a cell panel (**Figure 1B**) showed primary expression in monocytes, macrophages, dendritic cells, and polymorphonucleocytes, with some expression in colon epithelial and endothelial cells. Minimal expression was found in CD4/CD8 T cells or NK cells.

Pathway analysis represented extracellular matrix remodeling, complement regulation, and TNFA signaling, but revealed no pathways associated with adaptive immunity (**Table 1**).

**Conclusions:**

UC disease activity, as assessed by the endoscopic Mayo score, was strongly associated with tissue remodeling and molecules of innate immunity that were largely found in myeloid cells, colon epithelium, and endothelium. Cognate T cells were not dominant features of UC disease activity. These data suggest that the driver of ongoing UC activity is independent of the cognate T cell response.

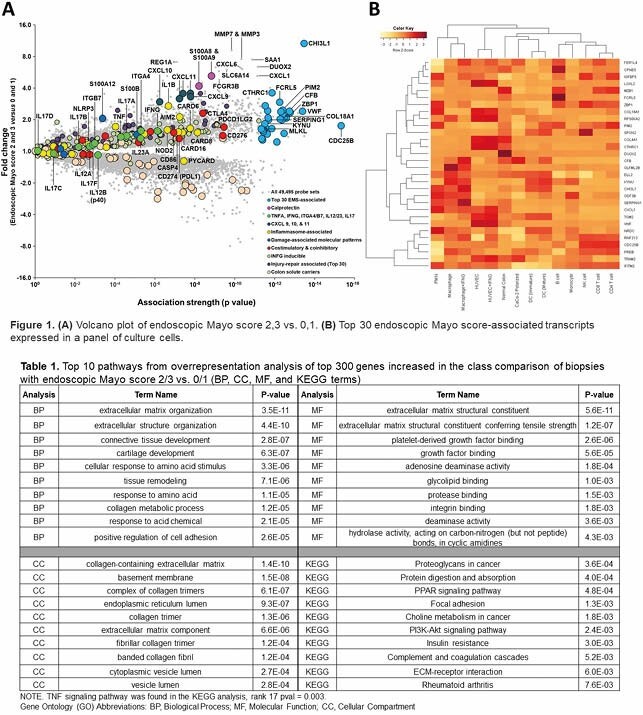

**Funding Agencies:**

None

